# Radiological signs of pulmonary congestion do not predict failed spontaneous breathing trial

**DOI:** 10.1186/2197-425X-3-S1-A1007

**Published:** 2015-10-01

**Authors:** ACP Antonio, AP Zanardo, PS Castro, MB Gazzana, C Teixeira, M Knorst

**Affiliations:** Universidade Federal do Rio Grande do Sul, Programa de Pós-Graduação em Ciências Pneumológicas, Porto Alegre, Brazil; Hospital de Clínicas de Porto Alegre, Porto Alegre, Brazil; Hospital Moinhos de Vento, Porto Alegre, Brazil

## Introduction

Both delayed and premature liberation from mechanical ventilation (MV) are associated with increased morbimortality. Positive pressure ventilation exerts beneficial effects in individuals with cardiogenic pulmonary edema; inspiratory fall in intra-thoracic pressure during spontaneous breathing trial (SBT), in its turn, may precipitate cardiac dysfunction through abrupt increase in venous return and in left ventricular afterload.

## Objectives

Determine the impact of radiological signs of pulmonary congestion prior to submission to SBT on weaning outcomes in a mixed ICU population.

## Methods

A prospective, observational study in an adult medical-surgical ICU. All enrolled patients met eligibility criteria for weaning from MV. Traqueostomized subjects were excluded. The primary end point was SBT failure, defined as inability to tolerate a T-piece trial during 30 to 120 minutes, in which case patient was not extubated. An attending radiologist applied a radiological score (RS)

## Results

There was a total of 170 SBTs procedures; SBT failure eventuated in 28 (16.4%). Nineteen patients (11.2%) had systolic heart failure (ejection fraction < 35%), 4 (2.4%) had chronic obstructive pulmonary disease (COPD) and 31 (18.2%) had been intubated due to respiratory sepsis. One hundred thirty-three patients (78.3%) were extubated at first attempt. RS was similar between SBT failure and success subjects (median 3 [2 - 4] vs 3 [2 - 4], p = 0.146), which means only intersticial lung congestion for both groups. Receiver operating characteristic (ROC) curves analysis demonstrated fail accuracy (area under curve [AUC] = 0.58) of CXRs prior to T-piece trial for discrimination between SBT failure and success individuals. There was no correlation between fluid balance in the 48 hours preceding SBT and RS (ρ = -0.13).

## Conclusions

Radiological findings of pulmonary congestion should not delay SBT indication since they did not predict greater probability of SBT failure in medical-surgical critically ill population.

**Figure 1:**
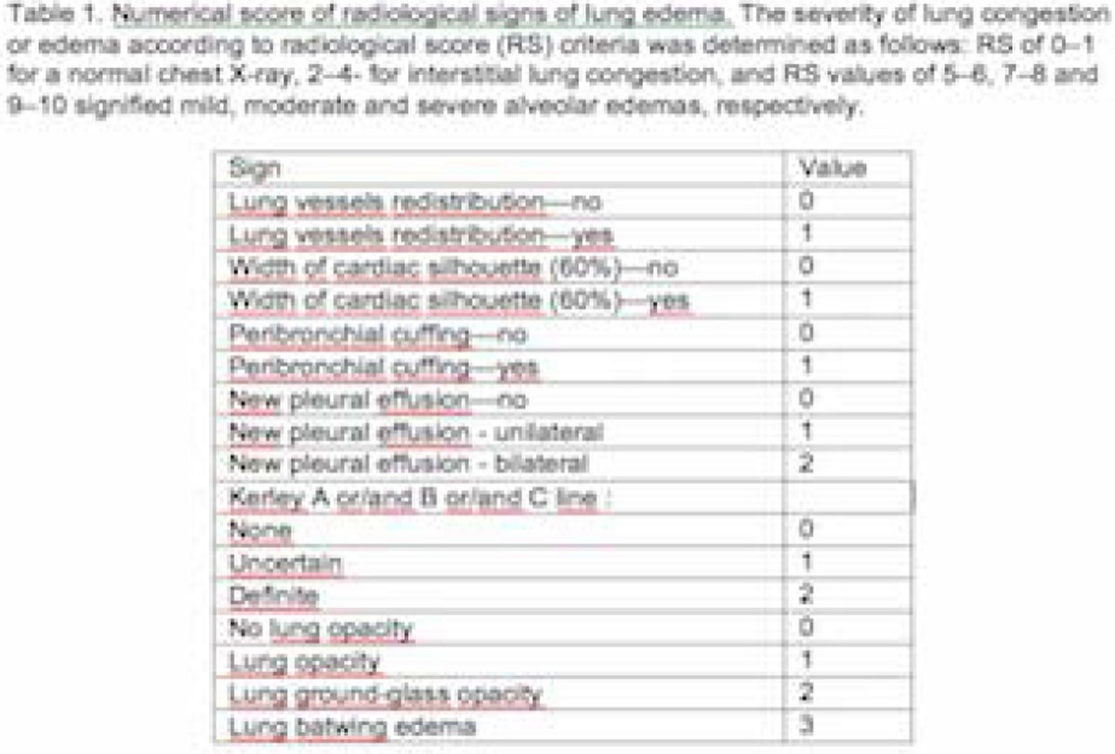
Figure 1
